# From epistemology to policy: reorienting philosophy courses for science students

**DOI:** 10.1007/s13194-022-00454-0

**Published:** 2022-03-29

**Authors:** Mark Thomas Young

**Affiliations:** TU Delft, Department of Technology, Policy and Values, Delft, The Netherlands

**Keywords:** Science in policy, Science advising, Expertise, Theoretical reason, Practical reason

## Abstract

Philosophy of science has traditionally focused on the epistemological dimensions of scientific practice at the expense of the ethical and political questions scientists encounter when addressing questions of policy in advisory contexts. In this article, I will explore how an exclusive focus on epistemology and theoretical reason can function to reinforce common, yet flawed assumptions concerning the role of scientific knowledge in policy decision making when reproduced in philosophy courses for science students. In order to address this concern, I will argue that such courses should supplement the traditional focus on theoretical reason with an analysis of the practical reasoning employed by scientists in advisory contexts. Later sections of this paper outline a teaching strategy by which this can be achieved that consists of two steps: the first examines idealized examples of scientific advising in order to highlight the irreducible role played by moral reasoning in justifying policy recommendations. The second employs argument analysis to reveal implicit moral assumptions in actual advisory reports that form the basis for class discussion. This paper concludes by examining some of the wider benefits that can be expected from adopting such an approach.

Throughout the last century, the kinds of questions scientists commonly address have changed. Historians, sociologists and philosophers have identified these forms of activity by different names, such as mandated science (Salter, 1988), regulatory science (Jasanoff, 1990), post-normal science (Funtowicz & Ravetz, 1993) and special interest science (Shrader-Frechette, 2014). Despite the differences between these concepts, all highlight the role scientists play as advisors and consultants in providing recommendations for practical and political decision-making. In the U.S this kind of scientific work underwent tremendous growth in the latter half of the century. Spurred on by what many perceived to be the successes of science during WW2, the postwar years saw a proliferation of scientific advisory committees tasked with providing recommendations to government on a wide range of issues. In the 1970’s, increasing public concern about the effects of industry on public health and the environment led to the creation of new government agencies such as the Environmental Protection Agency (EPA), which was tasked with utilizing scientific expertise to develop standards and regulations for industrial activity. Many of these regulations faced challenges in court, by industrial actors who drew on alternative sources of scientific expertise. By the end of the century, these changing relationships between government, industry and science had resulted in the emergence of a new range of consumers of scientific knowledge, such as government agencies, advisory committees, expert panels and interest groups, each of which functioned to bring scientists and political decision making closer together. Today, the demand for scientists to be involved in policy decision making continues to grow around the world. The increasing popularity of evidence-based policy, for example, reflects the widespread conviction that scientific knowledge forms a necessary component of rational political decision making.

Considering the significance of this shift in the kinds of work scientists perform and they role they play in society, we would be forgiven for assuming that science education too had changed – to reflect this shift by providing students of science with the tools to navigate advisory work in a reasonable, responsible and ethical manner. Yet unfortunately, this doesn’t seem to have been the case. As (Sienkiewicz et al., 2020) notes, ‘conventional academic training does not emphasize policy engagement as a central expectation in the scientific profession’. This neglect is also clearly evident in textbooks on the ethics of science, which have traditionally focused almost exclusively on research ethics at the expense of ethical concerns surrounding how research is used to support or guide policy decisions by scientific experts (Briggle & Mitcham, 2012: 17). It is perhaps not surprising then to find that not only do scientists often experience the kinds of questions they face in policy environments bewildering (Jasanoff, 1990: 94) or uncomfortable (Ravetz, 2006: 66), but also that, as we will see, many misunderstand the role that scientific knowledge can play in answering them.

It is here we might think that philosophy can make an important contribution to science education, by familiarizing students with the differences between practical and theoretical reasoning and exploring how the recommendations of advisory reports require different forms of justification than those utilized in support of theoretical conclusions in scientific research. After all, in many universities throughout the world, courses in philosophy form a mandatory component of degree programs in the natural sciences.^1^ Yet while philosophy of science may be uniquely poised to address those issues of policy advising which are so conspicuously absent from science education, it will only be able to do so by altering the way in which most courses in philosophy of science for science students (hereafter POSFSS) are approached. Existing literature on the role of philosophy in science education reveals a number of common themes regarding what the proper goals of such a course should be. Training in conceptual analysis is often emphasized, for example, to facilitate the clarification of scientific concepts (Martin, 1976) (Laplane et al., 2019). On the other hand, the need for a deeper and more critical understanding of the methods of science is also prioritized (Davson-Galle, 2004), sometimes by drawing upon perspectives from HPS (Grüne-Yanoff, 2014) (Kampourakis, 2017). Finally, the goal of cultivating a more nuanced and reflexive understanding of processes of reasoning in science by exploring the philosophical complexities of key themes such as induction, evidence and explanation, is often considered to be of central importance (Siegel, 1989) (Burbules & Lin, 1991). The common thread underlying each of these goals is the clear emphasis they place on epistemological aspects of science, which should not surprise us considering that, as Heather Douglas notes;

‘Most philosophers of science consider their work to belong to a subfield of epistemology, the study of knowledge, and as such are solely concerned with epistemological issues in science, such as the relationship between evidence and theory, the status of scientific theories, and the nature of scientific explanation. (Douglas, 2009: 44)’

Yet while the dominant focus on the epistemology of science has generated a range of fascinating and fruitful programs of philosophical research, there are important reasons why we might want to avoid restricting our focus to epistemology alone. Chief among these is the suggestion that the epistemological focus no longer captures the full range of philosophical issues that scientists must confront. Already by the early 1970’s for example, philosopher Jerome Ravetz argued that the traditional epistemological focus in the philosophy of science has;

‘…come down from periods when the conditions of work in science, and the practical and ideological problems encountered by its proponents were quite different from those of the present day. Science is no longer a marginal pursuit of little practical use carried on by a handful of enthusiasts; and it no longer needs to justify itself by a direct answer to the challenge of other fields of knowledge claiming exclusive access to truth. As the world of science has grown in size and in power, its deepest problems have changed from the epistemological to the social. (Ravetz, 1971: 9)

More recently, other philosophers have drawn attention to the way in which the dominant focus on the epistemology of abstract theoretical questions has functioned to exclude practical questions of how scientific knowledge can and should be used in policy environments from the traditional purview of philosophy of science (Douglas, 2009, 44) (Shrader-Frechette, 2014: 2). It is on the basis of such reasons that Heather Douglas for example, has called for a reorientation of the discipline of philosophy of science, away from an exclusive focus on epistemological issues and towards a conceptual and normative ‘examination of science as it functions in society in *all* of its aspects’ (Douglas, 2009, 22). Today, a growing number of philosophers of science are now heeding this call by turning their attention to the practical reasoning employed by scientists in advisory contexts.^2^

In this article, I want to explore how these changes hold particular significance for the role that philosophy of science plays in science education. In the first sections of this article I will outline reasons why I think this reorientation should be reflected in POSFSS courses, by supplementing the focus on epistemology with the analysis of practical reasoning utilized in advisory contexts. Later sections of this paper outline a teaching strategy by which this can be achieved that consists of two steps: the first examines idealized examples of scientific advising in order to highlight the irreducible role played by moral reasoning in justifying policy recommendations. The second employs argument analysis to reveal implicit moral assumptions in actual advisory reports that form the basis for class discussion. This paper concludes by examining some of the wider benefits that can be expected from adopting such an approach.

## Science, scientism and practical reason

In the opening pages of *Science and Ethics,* Bernard E. Rollin details the countless implicit and explicit ways in which what he terms “the standard line” – the idea ‘that science at most provides society with *facts* relevant to making moral decisions, but never itself makes such decisions’ (Rollin, 2006, 18), is taught to students throughout their scientific training. However, it’s not only in science textbooks and classes that one finds the standard line. For when the dominant epistemological focus of philosophy of science is reproduced in POSFSS courses, they too can be understood as reinforcing the standard line, by cultivating the impression that throughout the course of their professional lives, scientists engage exclusively in *theoretical reasoning*: reasoning which aims to justify descriptive conclusions about how the world actually is. Yet while common, this picture of scientific activity grossly misrepresents the reality which awaits many students who go on to have professional scientific careers. The increasingly central role of science in policymaking signals how scientists reason not only about knowledge, but also about action. This requires scientists to engage in *practical reasoning*, which unlike theoretical reason aims to establish normative conclusions about what should be done (Wallace, 2020). Practical reasoning is central to the activity of scientific advising. As Georg Brun and Gregor Betz note, ‘when experts derive policy recommendations in a scientific report, they set forth arguments for or against normative claims: they engage in practical reasoning, and so do the decision-makers who defend the choices they have made (Brun & Betz, 2016)’. While it is difficult to ascertain precisely how many scientists engage in practical reasoning of this kind, we gain some indications of the scope of this activity by observing how the majority of science addresses practical rather than theoretical goals. As Shrader-Frechette notes;

‘…in the United States, 75% of all science is funded by special interests in order to achieve practical goals, such as developing pharmaceuticals or showing some pollutant causes only minimal harm. Of the remaining 25 percent of US-science funding, more than half addresses military goals. This means that less than one-eighth of US-science funding is for basic science; roughly seven-eighths is for practical projects’ (Shrader-Frechette, 2014, 2)

We gain further insights into the extent to which scientists engage in practical reasoning by noting how advisory work is present in each of the three main areas that scientists find employment: academia, industry and government. Whereas consulting or advisory work represents a major component of the work performed by scientists for the government and industry, part time scientific consultancy work is nearly universal amongst academic faculty members (Sindermann & Sawyer, 1997: 251). As noted above however, dominant ideals of science as the disinterested pursuit of descriptive knowledge alongside the exclusion of practical reasoning from the training of scientists, have together reinforced the common idea that the reasoning employed by scientists is exclusively theoretical. The question of how to issue advice on normative questions of policy is therefore often experienced by scientists as a dilemma. There are two main ways in which scientists have attempted to address this dilemma by reconciling their training in theoretical reasoning with the demand of practical reason required by advisory work.

In the *first* place, scientists have sometimes attempted to distinguish within advisory work, the role they perform as scientists in engaging in theoretical reasoning from the role they perform as concerned citizens in arguing for particular normative conclusions. However, it is often impossible to maintain a clean separation of these roles in practice, as scientists are often unable avoid their status and authority as experts from lending credibility to the recommendations they provide (Douglas, 2009: 82) (Dessler & Parson, 2006: 39).

*Secondly,* scientists may attempt to refrain from engaging in practical reason altogether by restricting their activity as advisors to the communication of descriptive knowledge alone. The problem facing this strategy is that it often conflicts with the requirements of the job itself (Topp et al., 2020). As Carl Sindermann and Thomas Sawyer note in their guidebook for scientific consultancy, amongst the various activities consultants must perform in order to produce reports, such as gathering information or analysing and synthesising data, it is the final recommendations which ‘are probably the only parts of the report that will have the client’s full attention…and be the most meaningful, as they will indicate a course of action to resolve the problem that drove the client to employ a consultant in the first place’ (Sindermann & Sawyer, 1997: 157).

The difficulties facing such attempts to avoid practical reasoning as scientists in advisory contexts highlights the extent to which this now forms an unavoidable component of many scientific careers. Yet despite the fact that it is so common for scientists to engage in practical reasoning, my experience of teaching mandatory POSFSS courses over the past six years has revealed that this is not widely appreciated among first year science students. In the classroom, my attempts to illustrate the value and relevance of practical reasoning by exploring the complexities of policy questions has very often meet a stubborn and intransigent scientism: the view that ‘only science can provide us with knowledge or rational belief, that only science can tell us what exists and that only science can effectively address our moral and existential questions (Van Woudenberg et al., 2018). When confronted with the question of how we go about deciding whether or not to recommend restricting the exposure of children to EMF radiation for example, for many students, the answers are simple. After all, if we know the facts about a certain issue – shouldn’t they answer these questions for us? And if we don’t know the facts, then don’t we simply need to do more research?

It is not hard to see from where students get such ideas about the relation between science and policy. After all, the idea that scientific knowledge can compel policy decisions by revealing which actions should be taken remains a common feature of political discourse on scientific issues (Beck, 2011; Pielke Jr, 2007, 13). Ideas such as this undergird the common tendency to distinguish between governmental policies which “follow” the science, and those that do not,^3^ and reflect a longstanding and widespread perception in American political discourse that science represents an alternative and superior basis for decision-making than politics (Prewitt et al., 2012, 13). It is also promoted by scientists and public intellectuals, like Sam Harris, who argue that ‘science can, in principle, help us understand what we *should* do and *should* want – and therefore, what *other people* should do and should want in order to live the best lives possible’ (Harris, 2010, 28). These ideas are common not only in public discourse, but also in policymaking contexts themselves, where they are given expression by two models which aim to capture the role of scientific expertise in policymaking. The first is the rationalist model, which understands science to address pre-existing policy goals by identifying the *best* means by which they can be achieved (Keller, 2009, 29). The second is the positivist model, which understands science to not only identify the best means for achieving a policymaking goal, but also to play a role in determining those goals in the first place (Tagney, 2017). Today, this conception of the role science plays in policymaking remains common in many fields, such as environmental policy, where together these models are ‘still used as the default heuristic for evidence-based policymaking (Tagney, 2017) and where ‘recourse to the traditional notion of scientific objectivity as a powerful epistemological basis for resolving political debate remains in high currency’ (Keller, 2009, 39).

However, the idea that scientific expertise both can and should determine policy goals and the means by which they can be achieved appears to ignore important differences between theoretical and practical reasoning. In the remainder of this article, I want to explore why these differences are important and why challenging such scientistic attitudes towards policymaking should be included among the teaching goals of POSFSS courses. With this in mind, the following section introduces a teaching strategy which aims to highlight the differences between theoretical and practical reason through the analysis of real and idealized examples of advisory work.

## Teaching the argumentative analysis of advisory reports

The strategy I propose reflects the recent argumentative turn in policy analysis (cf. Hansson & Hadorn, 2016a) which involves approaching advisory reports as examples of informal argumentation that consist of one or more reasons arranged in support of a conclusion, and then providing them with basic philosophical tools necessary for evaluating the strength of those arguments. After introducing students to the distinction between normative claims which make recommendations concerning what *should* be done, and descriptive claims which aim to provide information concerning the way the world *is,* attention can be turned to what is perhaps the central rule of practical reasoning, that in order to be adequately justified, a normative claim must be supported by *both* descriptive and normative reasons (Betz, 2017). Insofar as policy recommendations establish normative claims then, they can be understood to require practical reasoning, which unlike theoretical reasoning requires the consideration of moral issues, such as what kinds of actions are right and therefore ought to be done.

The first step of this teaching strategy aims to illustrate how moral reasoning forms an irreducible component of the justification of policy recommendations. However, in order to demonstrate why this is the case, it is important to guard against the misconception that the demand for moral reasoning arises as a consequence of limitations in scientific knowledge. For this reason, it is helpful to employ thought experiments which bracket epistemological uncertainty, as illustrated by the following examples. The first case involves a scenario where there exist different means by which a policy goal can be achieved;

*Imagine a government wants to decide whether and how to regulate the use of a new pesticide. They assemble an expert panel of scientists to provide recommendations on how to proceed. Policymakers inform the panel that the only goal of the assessment is to reduce human harm. Now imagine that the expert panel possesses complete and accurate answers to any empirical questions they may consider relevant; they know the compound is less carcinogenic to humans than existing alternatives, they know that it causes less damage to the natural environment, that it costs less, and so on. How should they proceed?*

For many students it will most likely seem self-evident that the panel is justified in recommending the adoption of the new pesticide based on the information provided. However, the knowledge that a) policymakers desire to reduce human harm, and that b) this can be achieved by adopting the new pesticide over existing alternatives, together provide insufficient support for the claim that regulations *should* allow for the use of the new pesticide. As John Searle notes, ‘any means to a desirable end is desirable at least to the extent that it does lead to the end. But the problem is that in real life, any means may be and generally will be undesirable on all sorts of other grounds’ (Searle, 254). Adjudicating such conflicts often requires the use of moral reasoning. The panel might, for example, decide that *any* human harm resulting from pesticide use is morally unacceptable, and that the goal of reducing human harm is achieved best by prohibiting the use of both new and existing pesticides in order to facilitate a transition towards alternative agricultural techniques such as organic farming. As Kristin Shrader-Frechette notes, while environmental impact studies are often framed in ways that exclude such alternative means from consideration, this does not succeed in removing the need for moral reasoning in drawing normative conclusions;

‘consider the EPA ORBES assessment on which I worked. I am sure my co-workers, who were directed to find the best way of meeting energy demand in the basin would not claim to be making any ethical judgments about what ought to be done. Yet, by virtue of their own decision that coal and nuclear fission were the only viable options to be assessed, they did make an implicit evaluative judgment about whether society ought to consider alternatives such as on-site solar or nuclear fission’(EIA).

The second example examines a scenario in which scientists are able to identify only one means by which a policymaking goal can be achieved;

*Imagine that a team of scientists is assembled to provide recommendations on how to respond to the emergence of a new disease. Policymakers inform the panel that their only goal is the eradication of a disease within a community and ask them to provide a recommendation for how to proceed. In the course of their research, scientists determine that this goal can only be achieved by pursuing a policy of mandatory vaccination. What should the team recommend?*

Students again may feel that the panel is justified in recommending that mandatory vaccinations should be implemented. However, similar to the previous example, the knowledge that a) policymakers desire eradication of the disease and that b) this can only be achieved by mandatory vaccinations, together provides insufficient justification for the claim that we *should* make vaccinations mandatory. After all, just because a means may be the only way of achieving a goal does not mean that taking it is morally acceptable. This captures what many philosophers take to be an important feature of instrumental rationality, that;

‘the fact that a given means is necessary, relative to one’s ends is not a reason to take the means…suppose one intends end E and believes (truly) that E can only be achieved if one intends to do M. It appears that there are two ways in which one could revise one’s attitudes in response to these considerations, compatibly with the instrumental principle: one could form the intention to M, or one could abandon one’s original intention to E’ (Wallace, 2020).

Even in cases where there appears to be only one way in which a given policymaking goal can be achieved, the question of whether or not this goal *should* be pursued is a question that necessarily involves moral reasoning. A choice to recommend the implementation of mandatory vaccinations for example, implies that it is morally acceptable, given the circumstances, for the suspension of certain rights, such as the right to integrity which states that medical interventions must be based on an individual’s free and informed consent.

The preceding cases represent highly idealized examples of science advising. In the first place, they assume that policymaking is motivated by a single clearly defined goal. Secondly, and perhaps more importantly, they assume that scientists are in possession of complete and accurate knowledge of the consequences of pursuing different policy options. These idealizations serve the important function of drawing attention to the way moral reasoning represents an inherent feature of practical reason and does not arise as a result of limitations in knowledge. But of course, in the real word knowledge is *always* limited, and policymaking always occurs under conditions of uncertainty of varying kinds and degrees. This uncertainty applies not only to the situations motivating decision making in the first place, but also to the outcomes of the different options that are available to policymakers. It is exacerbated by the fact that in policymaking, decisions often have to be made quickly on the basis of severely limited information. These forms of uncertainty have important consequences for practical reasoning in advisory contexts because they increase the range of concerns that must be addressed by moral reasoning in the justification of policy recommendations. Arguments from inductive risk (cf. Douglas, 81) provide a simple illustration of one way in which epistemic uncertainty increases the scope of moral reasoning required to justify policy conclusions;

*Imagine that scientists are asked to provide recommendations in light of new data revealing a correlation between the consumption of a particular food additive and the development of certain forms of cancer. While the scientists deem the quality of evidence to be high, they are nonetheless aware that a correlation does not necessarily imply a causal connection. How should they proceed?*

In the case outlined above, recommending that the use of the additive should be discontinued implies that it is morally acceptable to overlook the possibility that the evidence is an unreliable indicator of a causal connection, and therefore that the risk of unnecessarily impacting employment, industry and the economy is a risk worth taking. The presence of epistemic uncertainty means that in forming policy recommendations scientists must decide whether a particular body of evidence is reliable enough to be acted on or not, and as Douglas notes ‘the judgment that some uncertainty is not important is *always* a moral judgment (Douglas, 2009, 85).

However, despite the irreducible role moral reasoning plays in justifying policy recommendations, these considerations are not always visible in the arguments scientists employ in the context of science advising. In fact, as Sven Ove Hansson and Gertude Hadorn note, ‘in policy debates, practical arguments – that is arguments for or against some policy option are often presented in incomplete and opaque ways. Important premises or steps of inference are often not expressed explicitly and their logical structure is intransparent’ (Hansson & Hadorn, 2016b). This observation applies particularly well to the arguments one finds in scientific advisory reports, which sometimes present their recommendations as based on descriptive scientific knowledge alone. In order to provide further illustration of the irreducible role played by moral reasoning in justifying policy recommendations, the second step of this teaching strategy employs argument analysis to reveal implicit moral assumptions in the reasoning employed in such reports. These assumptions are then intended to form the basis for later class discussions in which they can be subjected to critical scrutiny.

A useful model for this kind of argument analysis can be found in Kristin Shrader-Frechette’s analysis of the Rasmussen Report (WASH-1400), a 1975 report by the NRC on the safety of nuclear energy (Shrader-Frechette, 1980, 135–157). In her study, Shrader-Frechette examines the three main arguments the authors advance in support of the conclusion that the risks of reactor technology ought to be accepted and by doing so reveals how each argument contains only descriptive premises concerning the estimated magnitude of the risks involved. In order to satisfy the requirements of practical reason, Schrader-Frechette reconstructs each argument by identifying the following suppressed normative premises; a) all risks which have a low probability of occurring should be accepted, b) all risks which are lower than other risks already accepted by society are morally acceptable, and c) the estimated risks are insignificant compared to the expected economic benefits. As Shrader-Frechette notes, because each of these premises reflect to different extents, an implied utilitarian framework, the arguments in the report also ‘share the major weaknesses of this ethical theory. Some of these deficiencies include a general insensitivity to considerations of equity; a disregard for future generations; a tendency to equate desires with need; and an assessment of only quantifiable goods and bads’ (Shrader-Frechette, 1980, 149).

Shrader-Frechette’s analysis underscores how the argumentative analysis of advisory reports can provide teachers of POSFSS courses with a valuable opportunity to introduce students to major ethical theories in a practical and applied context. For this reason, such exercises can be understood to contribute to the development of skills important for deliberating on questions of policy, such as helping science students to develop what Jane Johnson calls an “ethical radar’; that is, a sensitivity that allows them to recognize moral issues as well as situations where values are in conflict’ (Johnson, 2010). Cultivating skills such as this has the potential to help students contribute more fruitfully to processes of deliberation surrounding normative aspects of policy questions by enabling them to identify a wider spectrum of potential moral concerns surrounding particular policy choices or recommendations.

Before concluding this section, I would like to highlight how the teaching strategy I have proposed provides teachers with a useful framework that has a number of specific benefits within the context of POSFSS courses. In the *first* place, it serves to underscore the relevance and practical importance of philosophy itself in science education, by highlighting how adequately addressing questions of policy requires skills of argumentation analysis alongside an understanding of the differences between theoretical and practical reasoning, both of which fall within the purview of philosophical rather than scientific training. *Secondly*, it encourages students to reflect critically on technocratic ideals in policymaking. As we have seen, this teaching strategy aims to demonstrate that unlike in the case of theoretical questions surrounding policy issues, scientists have no special authority to determine the answers to normative questions concerning what specific policies should be adopted (Dessler & Parson, 2006, 37). Insofar as we lack straightforward and universally accepted methods for determining what ends to pursue (Wallace, 2020), this strategy highlights reasons why these normative aspects of policy questions may be considered most appropriately addressed through democratic processes of public deliberation which include the perspectives and opinions of different actors and social groups which stand to be affected. *Thirdly*, following from these insights, this teaching strategy also serves to highlight a number of responsibilities that scientists have in advisory contexts, beyond providing accurate and truthful descriptions of scientific knowledge. These include the responsibility to help facilitate democratic decision processes on policy issues by attempting to clearly differentiate between theoretical and normative aspects of policy advice. As (Dessler & Parson, 2006, 42) note, while ‘it is not always possible to draw these distinctions perfectly cleanly…trying to do so to the extent feasible can bring large benefits. For individuals engaged in the policy debate…(it) will help to understand arguments that others are advancing, and provide a better basis for deciding whom to trust, to what degree, on what questions, and coming to an informed view of what decisions to favor. And for policy debate overall, pursuing such separation of questions is likely to reduce confusion and conflict, and provide a sounder basis for seeking courses of action that might gain broad support’. Other responsibilities include approaching the discussion of normative dimensions of policy questions in a way which recognizes the limits of scientific expertise by demonstrating an openness to the legitimacy of perspectives from outside the scientific community.

The frequency with which scientific experts have failed to meet these responsibilities in participatory governance schemes is well documented,^4^ and signals how the ideals traditionally associated with science may no longer be appropriate in an age where scientists are increasingly tasked with addressing questions of policy. As Ravetz notes;

the ‘objectivity’ that relies on an absence of people, judgments and values is no longer appropriate as an ideal. Instead, we should cultivate ‘integrity’ in science. For our dialogue on policy issues, we need participants to engage in a ‘negotiation in good faith’. each advances their case on the basis of their own clear and open perspectives and commitments (Ravetz, 2006, 75).

For many science students however, the image of work cultivated by such depictions may appear as more akin to politics than science, and likely represents a departure from the kind of activity they originally envisaged when they chose a career in natural science. This can be understood to be due in part to the way in which dominant images of scientific work have excluded such forms of activity by emphasizing, above all else, the epistemological dimensions of scientific practice. As I have argued throughout this paper however, altering such perceptions represents an important step towards preparing students to approach policy situations in a responsible manner.

## Conclusion

When I ask students in POSFSS courses to reflect on why they chose to study science over other disciplines, a common response is that what attracts them to science is that it deals with clear questions which can be answered concretely and definitively through empirical research, unlike disciplines such as philosophy which appear content to engage in discussion around questions that appear impossible to ever fully “solve” such as what actions can be considered right or just. It therefore comes as a surprise for many to learn that, not only do scientists address questions of the latter kind, but also *how much* scientific work falls into this category. Because it appears difficult, if not impossible for scientists to avoid addressing these kinds of issues directly in the practice of advisory work, and because such practices now form such a common element of the scientific profession, it is high time that we begin encouraging science students to recognize that scientific reasoning is not confined to the lab, and neither is it confined to exclusively epistemological issues. Instead, it can now be commonly found addressing political questions in the halls of government, the courtroom or public debates, where it often commands great authority. But with this authority comes the expectation that scientific expertise will be employed in a reasonable and responsible manner. For this to be possible it is essential that scientists recognize both the limits of scientific knowledge and the necessary role of moral reasoning in determining answers to questions of policy. Throughout this paper I have shown how this represents an opportunity by which philosophy of science can make an important contribution to the education and training of scientists. However, in order for philosophy of science to be able to address the unique challenges scientists face in an era of science for policy, it will require a reorientation away from the traditional focus on theoretical reason and towards the practical reason employed by scientists in advisory work.

## Footnotes

1 This is particularly true of Scandanavian countries where there are long established traditions of mandatory philosophy courses for all university degree programs. For further discussion of mandatory philosophy of science courses in Denmark, see also in this issue Green et al. (2021)

2 See for example, Shrader-Frechette (2014), Hansson and Hadorn (2016a), Steel (2015), Hansson (2013) and Betz (2017)

3 For recent critical analyses of the use of these ideas in relation to the Covid-19 pandemic, see Lowry (2020) and Yoder (2021)

4 See for example Wynne (1996), Healy (1999) and Turpenny et al. (2011)
